# Population pharmacokinetics and limited sampling strategy for therapeutic drug monitoring of mycophenolate mofetil in Japanese patients with lupus nephritis

**DOI:** 10.1186/s40780-022-00271-w

**Published:** 2023-01-09

**Authors:** Tomoko Mizaki, Hironobu Nobata, Shogo Banno, Makoto Yamaguchi, Hiroshi Kinashi, Shiho Iwagaitsu, Takuji Ishimoto, Yukiko Kuru, Masafumi Ohnishi, Ken-ichi Sako, Yasuhiko Ito

**Affiliations:** 1 Department of Pharmacy, Aichi Medical University Medical Center, 17-33 Nikkicho, Okazaki, Aichi 444-2148 Japan; 2 Department of Nephrology and Rheumatology, Aichi Medical University, 1-1 Yazakokarimata, Nagakute, Aichi 480-1195 Japan; 3 Medical Education Center, Aichi Medical University, 1-1 Yazakokarimata, Nagakute, Aichi 480-1195 Japan; 4 Department of Pharmacy, Aichi Medical University, 1-1 Yazakokarimata, Nagakute, Aichi 480-1195 Japan; 5 Department of Clinical Pharmacy, Nihon Pharmaceutical University, 10281 Komuro, Kitaadachigun Inamachi, Saitama, 362-0806 Japan

**Keywords:** Mycophenolate mofetil, Mycophenolic acid, Lupus nephritis, Limited sampling strategy, Population pharmacokinetics

## Abstract

**Background:**

Mycophenolate mofetil (MMF), a prodrug of the immunosuppressive agent mycophenolic acid (MPA), is difficult to administer because of the pharmacokinetic complexity of MPA. Although dosage adjustment according to the 12-h area under the concentration–time curve (AUC_0-12_) is thought to be desirable, multiple blood samplings for AUC calculation may pose a clinical challenge. A limited sampling strategy (LSS) would provide a solution; however, little is known about MPA pharmacokinetics in lupus nephritis patients, especially in those with Asian backgrounds, or few, if any, LSSs are reported for them.

**Methods:**

Thirty-four adult Japanese patients receiving MMF for lupus nephritis were examined retrospectively. MPA pharmacokinetics were investigated, and a PPK model was developed using Phoenix® NLME™ software. Single and double blood sampling strategies from Bayesian estimation using the PPK model and from multiple linear regression were compared. Tolerability was also evaluated.

**Results:**

In the pharmacokinetic analysis, renal function and serum albumin had significant effects on dose-normalized AUC_0-12_; and serum albumin, concomitant proton pump inhibitor (PPI) and iron/magnesium oxide did on dose-normalized maximum concentration. As a PPK model, a two-compartment model was developed with a transit absorption model and first-order elimination, in which creatinine clearance and serum albumin were covariates for MPA clearance. The double sampling strategy at 1 and 4 h by multiple linear regression showed the best agreement with the observed AUC_0-12_ (*r*^*2*^ = 0.885). Of the single sampling strategies, the one at 6 h by Bayesian estimation performed best (*r*^*2*^ = 0.769). The tolerability evaluation showed that correlations were suggested for gastrointestinal involvement.

**Conclusions:**

The present study developed the first PPK model of MPA for Japanese lupus nephritis patients. As for LSSs, a double sampling strategy at 1 and 4 h by multiple linear regression would work best; when only a single blood sampling is allowed, a strategy at 6 h by Bayesian estimation using the PPK model developed in this study would be best. The LSSs good enough for clinical use may facilitate safer, more effective, and individualized therapy.

**Supplementary Information:**

The online version contains supplementary material available at 10.1186/s40780-022-00271-w.

## Background

Mycophenolate mofetil (MMF) is a prodrug of the immunosuppressive agent mycophenolic acid (MPA). MMF is widely used in kidney and other organ transplantations and is also recommended as induction and maintenance therapy for lupus nephritis (LN), a renal manifestation of systemic lupus erythematosus [1]. Orally administered MMF is almost completely absorbed by the gastrointestinal tract and rapidly hydrolyzed to its active form MPA, which inhibits inosine 5'-monophosphate dehydrogenase (IMPDH), an enzyme involved in the de novo synthesis of guanosine in lymphocytes [2]. MPA is then primarily metabolized into the pharmacologically inactive mycophenolic acid glucuronide (MPAG) in the liver [3] and thereafter hydrolyzed back to MPA during enterohepatic recirculation (EHC), resulting in a second peak in MPA concentration [4].

In administering MMF, the pharmacokinetics of MPA should be taken into consideration. MPA has a narrow therapeutic window and exhibits wide inter- and intraindividual pharmacokinetic variability. The pharmacokinetics are affected by numerous factors, including renal function, liver function, serum albumin levels, concomitant drugs, and ethnic background [4–11]. Dosage reduction or drug withdrawal is sometimes necessary when adverse effects arise, such as infections or gastrointestinal symptoms [12–15]. Although a few studies have reported correlations between pharmacokinetic parameters and adverse effects [16–18], much remains unknown. Because of the administrative difficulties, adjusted dosage is desired rather than fixed dosage.

The 12-h area under the concentration–time curve (AUC_0-12_) for MPA reportedly correlates well with clinical outcomes [19, 20] and is considered to represent a valuable tool for adjusting MMF dosages. Although trough concentration could provide an alternative to AUC in general, such single-point measurements would not be appropriate in the case of MMF due to the second peak in MPA concentration [4, 21]. In patients receiving renal transplantation, a target range of 30–60 μg∙h/mL in AUC_0-12_ has been proposed in general, and therapeutic drug monitoring (TDM) using AUC_0-12_ has been suggested as a strategy for personalized treatment [22]. One clinical challenge with AUC_0-12_ is that calculation requires multiple blood samples. In MMF, 8–10 blood samples within 12 h would be suggested for ideal calculation, but in reality, blood collection at such a high frequency is not only costly and time-consuming, but also places a huge burden on patients. As a solution, a limited sampling strategy (LSS), which estimates AUC_0-12_ from a small number of samples, may be developed. For transplant patients receiving MMF, LSSs, varying from patient group to patient group, are commonly applied in TDM using AUC_0-12_ [23].

For Asian LN patients treated with MMF, however, the pharmacokinetic properties of MPA remain largely unexplored and few, if any, population pharmacokinetic (PPK) models, which may help in the development of LSS, appear to have been built. PPK models have been reported for transplant patients with various backgrounds [24–26], and a few have been described for autoimmune patients of different ethnicities [10, 27]; but these may not be applicable to Asian LN patients because of potential differences in MPA pharmacokinetics. While previous studies have reported that a range of 30–45 μg∙h/mL in AUC_0-12_ is associated with good clinical outcomes [28], an LSS appears less likely to be available for Asian LN patients.

This study aimed to develop an LSS for Japanese LN patients receiving MMF, in the hope that the LSS would help facilitate safer, more effective, and individualized therapy for such patients. For this purpose, MPA pharmacokinetics after oral administration of MMF were investigated first, and then a PPK model was constructed to describe the pharmacokinetics. From the resulting model, an LSS was developed to estimate AUC_0-12_ from single or double blood sampling. For comparison, two distinct approaches were employed in developing an LSS: maximum a posteriori Bayesian estimation (MAP-BE) and multiple linear regression (MLR).

## Methods

### Patients and data collection

Participants comprised all Japanese patients ≥ 18 years old who received MMF (CellCept®; Chugai Pharmaceutical Group Co., Tokyo, Japan) for treatment of LN at Aichi Medical University Hospital (Nagakute, Japan) between March 2015 and June 2022 and for whom data were available on multiple blood samples necessary to make a pharmacokinetic profile.

From the electronic medical records, the following demographic and clinical data of patients were retrospectively collected: sex; age; body weight; laboratory data, including serum albumin, serum creatinine, urine protein/creatinine ratio (UPC), alanine aminotransferase (ALT), aspartate aminotransferase (AST), total bilirubin (TBIL), and C-reactive protein (CRP); dose of MMF; dose of concomitant prednisolone; other concomitant medications, including tacrolimus, proton pump inhibitors (PPIs), iron/magnesium oxide, and non-steroidal anti-inflammatory drugs (NSAIDs); and adverse effects. Estimated glomerular filtration rate (eGFR) was calculated from serum creatinine levels using a new 3-variable Japanese equation [29], and creatinine clearance (CLcr) was calculated using the Cockcroft-Gault equation [30]. As adverse effects of MMF, gastrointestinal involvement, infection, leukopenia, and alopecia were reported. Of these, gastrointestinal symptoms and infections were examined as major adverse effects [12–15].

MMF was administered twice a day, every 12 h. In a steady state after multiple administration, blood samples were taken at 9 time points during the dosing interval: right before administration (C_0_) and 0.5, 1, 2, 3, 4, 6, 8, and 12 h after administration (C_0.5_, C_1_, C_2_, C_3_, C_4_, C_6_, C_8_, and C_12_, respectively). MPA concentrations were measured using enzyme immunoassay on cobas® 6000 c501 (Roche Diagnostics K.K.; Tokyo, Japan) with a commercially available cobas® MPA Kit (Roche Diagnostics K.K.; Tokyo, Japan). The lower limit of quantification was 0.40 μg/mL.

### Pharmacokinetic analysis

The parameters used for pharmacokinetic analysis were maximum MPA concentration within a dosing interval of 12 h (Cmax), time to reach maximum MPA concentration (Tmax), and AUC_0-12_. Cmax and Tmax were derived from the MPA concentration-versus-time profiles of patients. AUC_0-12_ was calculated from MPA concentrations at the 9 time points of C_0_ to C_12_, using the linear trapezoidal method. Dose-normalized Cmax and dose-normalized AUC_0-12_ were also calculated. Correlations were investigated between AUC_0-12_ and MMF dose, as well as between AUC_0-12_ and C_0_ MPA concentration, using the Spearman rank correlation test, where AUC_0-12_ was regarded as a dependent variable and the dose or concentration as an independent variable.

From a clinical point of view, we performed group comparisons to investigate the effect of biochemical factors on the pharmacokinetic parameters of MPA, as the previous study [31] did. The patients were divided into two groups by eGFR (< 81 mL/min versus ≥ 81 mL/min), and also by serum albumin level (< 3.5 g/dL versus ≥ 3.5 g/dL). Similarly, for the effect of concomitant drugs, patients were split into two groups by the presence or absence of each of the following drug administrations: tacrolimus, PPI, and iron/magnesium oxide. In all group comparisons, differences in Tmax, dose-normalized Cmax, and dose-normalized AUC_0-12_ were examined using the Mann–Whitney U test.

All statistical analyses of pharmacokinetics were performed using easy R (EZR) version 1.54 [32].

### PPK model development and validation

All analyses for PPK model development and validation, as well as subsequent LSS development and validation, were performed using Phoenix® NLME™ software (version 8.1; Pharsight, Mountain View, CA, USA) and EZR version 1.54 [32]. For the estimation algorithm, the first-order conditional estimation-extended least-squares estimation method was employed. For the overall procedure, Bonate [33], Owen and Fiedler-Kelly [34], Gabrielsson and Weiner [35], and *Guidance for Industry: Population Pharmacokinetics* [36] were referred to.

The PPK model was developed in a step-by-step manner. First, in the search of a base model, one-, two- and three-compartment models with first-order elimination were tested. These models were parameterized in terms of absorption rate constant (Ka), central volume of distribution (V_1_), central compartment clearance (CL), peripheral volume of distribution (V_2_), and inter-compartmental clearance (Q). Because absolute bioavailability (F) was not to be assessed in cases of oral administration, V_1_/F, CL/F, V_2_/F, and Q/F were considered to correspond to V_1_, CL, V_2_, Q, respectively. Using the resulting base model, the intra-individual variability of MPA concentration was examined by comparing additive, proportional, and mixed (additive plus proportional) residual error models. Inter-individual variability was examined using the exponential error model.

As the next step, covariate models were developed and the following variables were tested as covariates: age, sex, body weight, eGFR, CLcr, serum albumin, UPC, ALT, AST, TBIL, CRP, dose of concomitant prednisolone, and concomitant drugs of PPI and iron/magnesium oxide. In addition, an EHC model was also examined in reference to a previous study [27]. Finally, whether the MPA absorption process was well described was examined by comparing models with and without lag time. For the same purpose, transit models with different numbers of compartments (1 to 7) were also compared.

In these steps of model development, model selection decisions were made based primarily on the likelihood ratio test. In the test, objective function values (OFVs) in nested models were compared using the χ^2^ test. If the difference between models resulted in OFV greater than the critical value (i.e., 6.63 units when models differ by 1 degree of freedom), the more complex model was considered to be significantly better (*p* < 0.01). Subsequently, relative standard errors (RSE) were calculated for the evaluation of adequacy, and the condition number (i.e. the ratio of the absolute highest and lowest eigenvalues) for overparameterization. In both calculations, a smaller value was considered to be better. As another way to see adequacy, goodness-of-fit plots were performed. Goodness-of-fit plots were examined for observed concentrations versus individual predicted concentrations, observed concentrations versus population-predicted concentrations, conditional weighted residuals versus time since the dose, and conditional weighted residuals versus population-predicted concentrations [37, 38].

For validation, models in the development process were examined using the prediction-corrected visual predictive check (pcVPC, 1000 simulations) [39]. The precision of parameter estimates was assessed using the bootstrap method (1000 bootstrap samples).

### LSS development and validation

From a clinically practical point of view, we developed single and double blood sampling strategies. In the development, we employed two distinct methods: an MAP-BE approach using the PPK model and an MLR approach [40]. In the MAP-BE approach, from one or two observed MPA concentrations, the remaining values from C_0_ to C_12_ were estimated based on the newly developed PPK model. From the observed and estimated MPA concentrations, the predicted value of AUC_0-12_ (AUC_predicted_) was calculated. In the MLR approach, multiple linear regression analysis was performed between one or two observed values of MPA concentration (independent variables) and AUC_observed_ (dependent variable), which was calculated in the same way as AUC_0-12_. Using the resulting MLR equation, AUC_predicted_ was calculated from the one or two observed values.

For validation, adjusted coefficients of determination (*r*^2^) were used to examine the regression level of AUC_predicted_ and AUC_observed_. Accuracy and precision of prediction were evaluated at each time point from C_0_ to C_12_, using mean prediction error (MPE), 95% confidence interval (CI) of MPE, and root mean squared percentage error (RMSPE). Acceptance ranges were set as ≤  ± 10% in MPE and ≤ 25% in RMSPE [40]; CI of MPE was considered to be acceptable when it crossed the value of 0. In the equations below, N represents the number of pairs of AUC_predicted_ and AUC_observed_.

$$\mathrm{MPE}\;(\%)=\frac1{\mathrm N}\cdot\;{\textstyle\sum_{i=1\;}^{\mathrm N}}\left(\frac{{\mathrm{AUC}}_{\mathrm{predicted}}-{\mathrm{AUC}}_{\mathrm{observed}}}{{\mathrm{AUC}}_{\mathrm{observed}}}\right)\times100$$

$$\mathrm{RMSPE}\;(\%)=\sqrt{\frac1{\mathrm N}}\cdot{\textstyle\sum_{i=1}^{\mathrm N}}\left(\frac{{\mathrm{AUC}}_{\mathrm{predicted}}-{\mathrm{AUC}}_{\mathrm{observed}}}{{\mathrm{AUC}}_{\mathrm{observed}}}\right)^2\times100$$

### Tolerability evaluation

As major adverse effects, gastrointestinal symptoms and infections were examined. Using the Mann–Whitney U test, patients with and without gastrointestinal involvement were compared in terms of dose, dose/body weight, C_0_, Cmax, AUC_0-12_, AUC_0-12_/dose, and AUC_0-0.5_; and so were patients with and without infection. If the correlation between a parameter and either of the major adverse occurrences was suggested, receiver operating characteristic (ROC) curve analysis was performed for the parameter, to determine the optimal cutoff. Statistical analyses were performed using EZR version 1.54 [32].

## Results

### Patients

Thirty-four Japanese ≥ 18 years old who received MMF for treating LN were enrolled. They received MMF at doses of 250–1,000 mg every 12 h twice a day, as prescribed in the package insert. Demographic and clinical characteristics of the patients are shown in Table 1.

**Table 1:** Demographic characteristics of patients (*n* = 34)

Patient characteristics	Median (IQR) or number	
Sex		
Male	6	
Female	28	
Age (years)	39.0	(26.3–51.8)
Body weight (kg)	54.7	(47.2–58.6)
Serum albumin (g/dL)	3.6	(2.8–3.9)
Serum creatinine (mg/dL)	0.66	(0.54–0.79)
Estimated GFR (mL/min)	77.4	(60.3–93.1)
Creatinine clearance (mL/min)	103.6	(71.1–125.0)
Urine protein/creatinine ratio (g/gCre)	0.25	(0.14–1.62)
Alanine aminotransferase (U/L)	16.0	(13.0–20.0)
Aspartate aminotransferase (U/L)	17.0	(13.3–22.0)
Total bilirubin (mg/dL)	0.56	(0.43–0.66)
C-reactive protein (mg/dL)	0.06	(0.04–0.15)
Dose of MMF (mg/day)	1500	(1500–1688)
Dose of MMF/body weight (mg/day/kg)	27.9	(24.1–33.2)
No. of patients receiving concomitant drugs		
Prednisolone	34	
Dose of prednisolone (mg)	10	(8–30)
Tacrolimus	8	
Proton pump inhibitor	22	
Iron/magnesium oxide	5	
NSAIDs	3	

*IQR* interquartile range, *GFR* glomerular filtration rate, *NSAIDs* nonsteroidal anti-inflammatory drugs

In 33 patients, data for blood samples taken at all 9 time points (C_0_ to C_12_) were available, making full pharmacokinetic profiles. In 1 patient, data for only 5 time points (C_0_, C_1_, C_2_, C_3_, and C_4_) were obtained. The data set contained 302 MPA plasma concentrations obtained from 34 patients, which were used for PPK model construction. For pharmacokinetic analysis and LSS development, full profiles of the 33 patients were used.

### Pharmacokinetic analysis

**Fig. 1:**
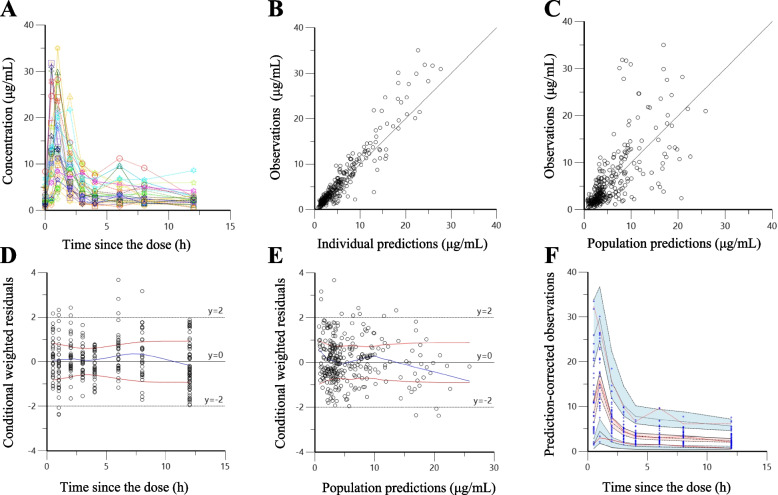
Concentration versus time, goodness-of-fit plots of the PPK final model, and a visual predictive check for the final model. **A** Concentration versus time profiles of MPA after administration. **B**–**E** Goodness-of-fit plots of the final model. **B** Scatter plots of observed concentrations versus individual predicted concentrations. **C** Observed concentrations versus population-predicted concentrations. **D** Conditional weighted residuals versus time since the dose. **E** Conditional weighted residuals versus population-predicted concentrations. **F** Prediction-corrected visual predictive check for the final model, where blue circles indicate observed concentrations, red lines represent the observed median and 5th and 95th percentiles, and shaded regions indicate 90% CIs for the simulations (orange regions indicate median, while blue regions indicate the 5th and 95th percentiles)

For the 34 patients, median (first quartile—third quartile) C_0_ MPA concentration was 1.8 (1.1–2.9) μg/mL; median Cmax was 16.7 (10.9–26.9) μg/mL; median Tmax was 1.0 (1.0–1.0) h; and median AUC_0-12_ was 51.1 (41.1–72.7) μg∙h/mL. In 32 of the 33 patients with a full profile, the second peak was observed: the median second peak of MPA concentration was 3.3 (2.3–5.6) μg/mL, and the median period from administration of MMF to the second peak was 8 (6–9) h (Fig. 1A). AUC_0-12_ did not correlate with MMF dose (*r*^2^ = 0.14) (Additional file 1A), but did correlate with C_0_ MPA concentration (*r*^2^ = 0.79) (Additional file 1B).

**Fig. 2:**
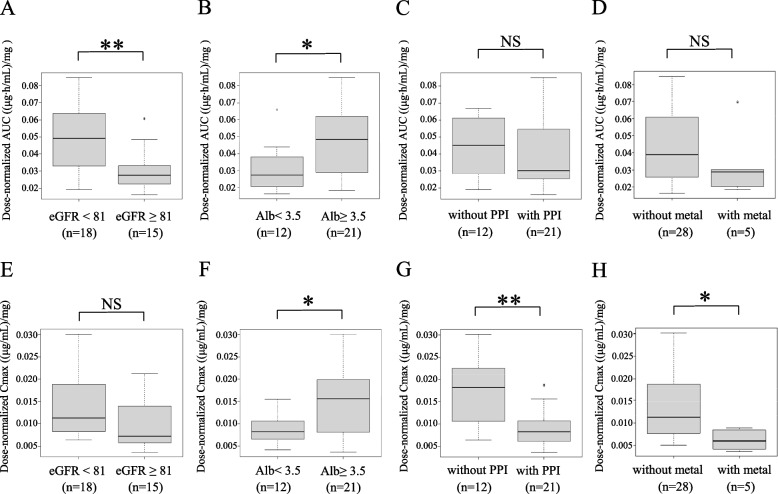
Effects of pharmacokinetic parameters. **A** Dose-normalized MPA-AUC of patients divided by eGFR level (< 81 mL/min vs ≥ 81 mL/min). **B** Dose-normalized MPA-AUC of patients divided by serum albumin level (< 3.5 g/dL vs ≥ 3.5 g/dL). **C** Dose-normalized MPA-AUC of patients with presence or absence of PPI. **D** Dose-normalized MPA-AUC of patients with presence or absence of metal (iron/magnesium oxide). **E** dose-normalized Cmax of patients divided by eGFR level (< 81 mL/min vs ≥ 81 mL/min). **F** Dose-normalized Cmax of patients divided by serum albumin level (< 3.5 g/dL vs ≥ 3.5 g/dL). **G** Dose-normalized Cmax of patients with presence or absence of PPI. H) Dose-normalized Cmax of patients with presence or absence of metal (iron/magnesium oxide). **: *p* < 0.01, *: *p* < 0.05, NS: not significant

The effects of eGFR and serum albumin on MPA pharmacokinetics were observed in dose-normalized AUC_0-12_: patients with eGFR < 81 mL/min (*n* = 18) showed significantly higher dose-normalized AUC_0-12_ than those with eGFR ≥ 81 (mL/min) (*n* = 15) (Fig. 2A), and patients with serum albumin < 3.5 g/dL (*n* = 12) showed significantly lower dose-normalized AUC_0-12_ than those with serum albumin ≥ 3.5 g/dL (*n* = 21) (Fig. 2B). On dose-normalized Cmax, serum albumin had significant effects: patients with serum albumin < 3.5 g/dL displayed lower dose-normalized Cmax than those with serum albumin ≥ 3.5 g/dL (Fig. 2E F). Neither eGFR nor serum albumin had significant effects on Tmax.

The effects of concomitant administration of PPI nor iron/magnesium oxide were not observed in dose-normalized AUC_0-12_ (Fig. 2C D), but in dose-normalized Cmax. It was significantly lower in patients with concomitant PPI (*n* = 21) than in those without it (*n* = 12) (Fig. 2G); and likewise in patients with iron/magnesium oxide (*n* = 5), compared to those without it (*n* = 28) (Fig. 2H). On Tmax, concomitant iron/magnesium oxide showed an effect: patients receiving iron/magnesium oxide (*n* = 5) displayed a significantly increased Tmax compared to those without such treatment (*n* = 28).

### PPK model development and validation

**Fig. 3:**
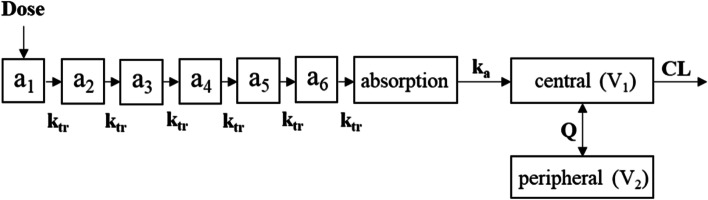
Schematic diagram of the final population pharmacokinetic model. Transit compartments (a_1_-a_6_), absorption compartment, central compartment, and peripheral compartment for MPA. Ktr, rate constant of transit compartment [Ktr = (n + 1) / MTT, where n is the estimated number of transit compartments prior to the absorption compartment and MTT is the mean transit time into the first depot compartment]; Ka, absorption rate constant; V_1_, central compartment volume of distribution; V_2_, peripheral compartment volume of distribution; CL, central compartment clearance; Q, inter-compartmental clearance

The structure of the final PPK model that adequately described MPA pharmacokinetics of the 34 patients is given in Fig. 3. As a base model, a two-compartment linear model with first-order elimination was employed. Intra-individual variabilities were best described by a proportional residual error model. Then models with different covariant patterns were developed. Of these models, the one including all the variables significant in the pharmacokinetic analysis, i.e. CLcr and serum albumin for CL and concomitant PPI and iron/magnesium oxide for V1, was best in OFV. The model with the four variables, however, was suspected for overparameterization, and therefore was compared to the other candidate model, which included CLcr and serum albumin alone as covariates. The comparison found that, although the model with the four covariates had a significantly better OFV, the model with the two covariates had a substantially decreased value in other areas. RSE (%) for V_1_ decreased from 32.71 in the model with the four variates to 17.71 in the model with the two covariates, RSE (%) for IIV V_1_ from 52.92 to 28.18, and the condition number from 5128.3 to 1848.7, indicating overall improvement. In addition, the confidence interval of 1000 Bootstrap for V1 was further examined. While it was 32.41–168.41 in the model with the four covariates, it was 13.43–32.75, decreased by about 85%, in the model with the two covariates, showing that the reliability of the estimates was much improved by excluding PPI and iron/magnesium oxide from covariates (Table 2 and Additional file 2). The model with CLcr and serum albumin alone as covariates was thus adopted for the final model. In the next step, transit compartments were added one at a time, up to seven, and each model was examined. The model with lag time and the EHC model were also examined. The model with 6 compartments significantly improved the model fit and better described the absorption process. The model with lag time or the EHC model did not improve the model fit.

**Table 2:** Parameter estimates and bootstrap results of the final model

Parameter	Final model		1000 Bootstrap Results		
	Estimate	RSE (%)	Estimate	95% LLCI	95% ULCI
Population mean					
V_1_ (L)	22.95	17.71	21.99	13.43	32.75
CL (L/h)	13.15	7.87	12.88	9.49	15.12
Ka (h^−1^)	2.98	8.90	2.96	2.23	3.87
V_2_ (L)	336.03	26.11	350.24	162.42	668.78
Q (L/h)	26.44	7.90	26.56	22.07	30.97
MTT (h)	0.45	12.32	0.46	0.34	0.60
Effect CLcr on CL	0.78	20.22	0.81	0.46	1.28
Effect Alb on CL	-0.88	16.10	-0.92	-1.34	-0.51
Interindividual variability					
IIV V_1_ (CV%)	86.96	28.18	87.80	22.85	152.75
IIV CL (CV%)	32.23	25.61	32.06	23.70	40.42
IIV MTT (CV%)	58.50	33.37	57.13	31.81	82.45
Residual variability					
Proportional error (CV%)	34.09	6.92	33.86	29.86	38.55

*RSE* relative standard error, *95% LLCI* lower limit of the 95% confidence interval, *95% ULCI* upper limit of the 95% confidence interval, *V*_*1*_ central volume of distribution, *CL* central compartment clearance, *Ka* absorption rate constant, *V*_*2*_ peripheral volume of distribution, *Q* inter-compartmental clearance, *MTT* mean transit time, *CLcr* creatinine clearance, *Alb* serum albumin, *IIV* interindividual variability

CL (L/h) = 13.15 × ($\frac{\mathrm{CLcr}}{{\mathrm{CLcr}}_{\mathrm{median}}}$)^0.78^ × ($\frac{\mathrm{Alb}}{{\mathrm{Alb}}_{\mathrm{median}}}$)^−0.88^ × exp(ηCL)

CLcr_median_ was 6.2 L/h, Alb_median_ was 3.6 g/dL

V1 (L) = 22.95 × exp(ηV)

All goodness-of-fit plots showed the high predictive performance of the final model, with no systematic deviations observed (Fig. 1B-E). The relative errors were comparable across all concentration ranges (Fig. 1E, Additional file 3). The prediction-corrected visual predictive check is shown in Fig. 1F. The observed median and 5th and 95th percentiles were located inside the 90% CI of the simulated data. Parameter estimates by the bootstrap method closely matched the means of corresponding parameter estimates from the final model, confirming the stability of the final model (Table 2).

### LSS development and validation

Table 3 shows the best four predictive performances for each of single and double blood sampling strategies under the MAP-BE approach (using Bayesian estimation), and the MLR approach (using multiple linear regression analysis). For both approaches, double sampling strategies were more accurate than single ones. In the MAP-BE approach, the MPE and RMSPE values were all within the clinically acceptable ranges of ≤ 10% and ≤ 25%, respectively; and the CIs of MPE crossed the value of 0, except for single sampling at C_0_, single sampling at C_4_, double sampling at C_1_ and C_3_, and double sampling at C_1_ and C_4_. In the MLR approach, the RMSPE values of the single sampling strategies were all larger than the acceptable range, while those of the double sampling strategies were all within the acceptable range. The MPE values were all within the acceptable range and the CIs of MPE all crossed the value of 0.

**Table 3:** Best performing single and double sampling strategies for estimation of AUC_0-12_ of MPA

**(A) Bayesian estimator**						
Sampling points	*r*^2^	*p*	MPE (%)	95% LLCI	95% ULCI	RMSPE (%)
C_4_	0.713	< 0.001	-6.53	-15.23	2.18	25.04
C_8_	0.746	< 0.001	-2.58	-9.99	4.84	20.75
C_0_	0.754	< 0.001	-11.86	-18.23	-5.48	21.30
C_6_	0.769	< 0.001	-0.72	-7.51	6.08	18.89
C_6_, C_12_	0.851	< 0.001	-2.46	-8.72	3.80	17.56
C_6_, C_8_	0.854	< 0.001	0.15	-5.38	5.69	15.36
C_1_, C_3_	0.860	< 0.001	-10.49	-16.72	-4.25	20.25
C_1_, C_4_	0.883	< 0.001	-12.83	-18.43	-7.22	20.17
**(B) Linear regression**						
Model equation	*r*^2^	*p*	MPE (%)	95% LLCI	95% ULCI	RMSPE (%)
AUC_0-12_ = 11.2C_0_ + 31.6	0.654	< 0.001	7.66	-1.98	17.29	27.83
AUC_0-12_ = 11.4C_8_ + 21.8	0.679	< 0.001	7.10	-2.89	17.09	28.63
AUC_0-12_ = 8.3C_6_ + 26.8	0.695	< 0.001	6.66	-2.86	16.18	27.26
AUC_0-12_ = 10.6C_4_ + 21.5	0.702	< 0.001	6.06	-3.53	15.65	27.32
AUC_0-12_ = 6.5C_0_ + 6.9C_4_ + 18.9	0.837	< 0.001	4.03	-3.46	11.53	21.20
AUC_0-12_ = 5.2C_6_ + 6.8C_8_ + 16.8	0.840	< 0.001	4.12	-2.75	10.99	19.53
AUC_0-12_ = 1.4C_1_ + 9.2C_8_ + 9.2	0.844	< 0.001	4.85	-2.30	12.00	20.43
AUC_0-12_ = 1.4C_1_ + 8.7C_4_ + 7.8	0.885	< 0.001	2.50	-2.74	7.73	14.75

*95% LLCI* lower limit of the 95% confidence interval of MPE, *95% ULCI* upper limit of the 95% confidence interval of MPE, *MPE* mean prediction error, *RMSPE* root mean squared percentage error

**Fig. 4:**
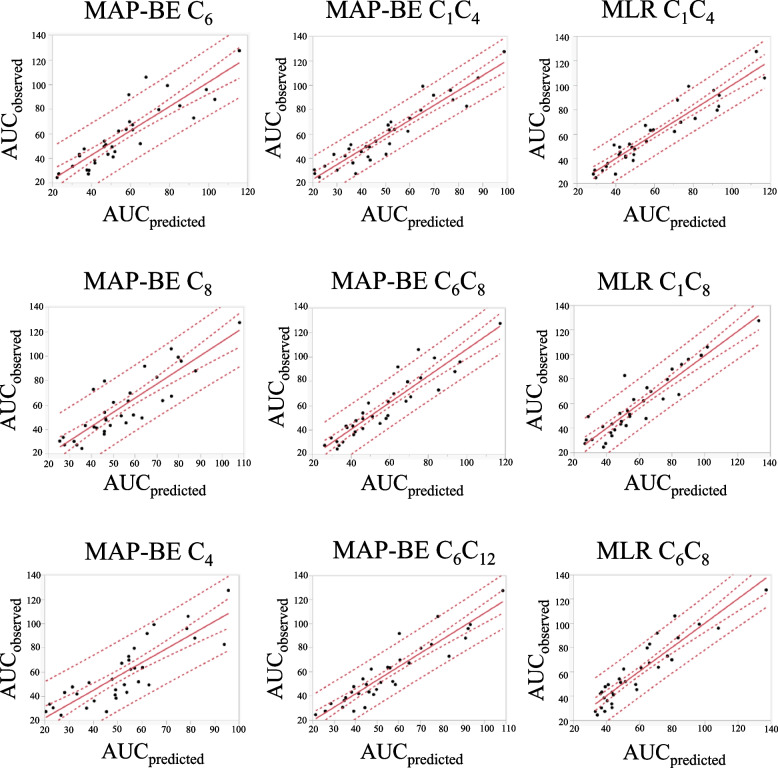
Observed versus predicted AUC_0-12_ of MPA. The straight line represents the identity line. The outer dot line indicates the limit of confidence interval, and the inside dot line indicates the limit of credible interval. MAP-BE, maximum a posteriori Bayesian estimation; MLR, multiple linear regression; AUC_observed_, AUC_0-12_ calculated from MPA concentrations at the 9 time points of C_0_ to C_12_, using the linear trapezoidal method; AUC_predicted_, the predicted value of AUC_0-12_

The best agreement with AUC_observed_ was achieved by the double blood sampling strategy of C_1_ and C_4_ by the MLR approach, where *r*^*2*^ was 0.885, MPE was 2.50% (95%CI, -2.74–7.73), and RMSPE was 14.75%. Of the single sampling strategies, C_6_ sampling strategy by the MAP-BE approach displayed the best agreement with the AUC_0-12_, where *r*^*2*^ was 0.769, MPE was -0.72% (95%CI, -7.51–6.08), and RMSPE was 18.89%. The estimation accuracy was found to be good at near the target value of AUC_0-12_ (30–45 μg∙h/mL) (Fig. 4).

### Tolerability evaluation

The results for major adverse occurrences (gastrointestinal involvement and infection) are shown in Table 4. Gastrointestinal involvement occurred in 10 of the 33 patients, and diarrhea, vomiting, and epigastric pain were observed. Infections occurred in 14 patients, and upper respiratory infection, fever, pharyngitis, bronchitis, oral herpes zoster, Malassezia, and Aspergillus were observed. No case of cytomegalovirus infection was observed. For gastrointestinal involvement, correlations were suggested for three parameters: AUC_0-0.5_, Cmax and AUC_0-12_ were significantly higher in patients with gastrointestinal involvement (*n* = 10) than those without gastrointestinal involvement (*n* = 23) (*p* < 0.01, *p* < 0.05 and *p* < 0.05, respectively). No correlation was suggested between infection and any of the examined parameters.

**Table 4:** Relationship between pharmacokinetic parameters of MPA and development of adverse effects

		*n*	Dose	Dose/body weight	C_0_	Cmax	AUC_0-12_	AUC_0-12_ / Dose	AUC_0-0.5_
			(mg)	(mg/kg)	(μg/mL)	(μg/mL)	(μg∙h/mL)	(μg∙h/mL/mg)	(μg∙h/mL)
Gastrointestinal involvement	+	10	1500 (1500–1500)	28.3 (25.8–30.9)	1.9 (1.3–3.1)	26.3* (15.4–29.7)	63.4* (53.8–89.4)	0.049 (0.031–0.062)	6.3** (4.2–7.9)
	−	23	1500 (1500–1875)	227.6 (23.6–35.0)	1.8 (1.2–2.8)	12.3 (8.7–19.7)	45.3 (34.8–68.4)	0.030 (0.024–0.052)	2.9 (1.0–3.7)
Infection	+	14	1500 (1500–1875)	28.5 (26.3–33.2)	1.6 (1.2–3.2)	17.0 (10.9–23.5)	49.7 (41.3–77.8)	0.028 (0.024–0.047)	3.3 (2.5–3.9)
	−	19	1500 (1500–1625)	27.3 (21.9–34.6)	1.9 (1.2–2.6)	12.8 (10.4–27.7)	51.1 (40.8–68.4)	0.036 (0.030–0.061)	3.7 (1.1–6.6)

Values represent the median (IQR). * *p* < 0.05, **p < 0.01

Gastrointestinal symptoms: diarrhea, vomiting, and epigastric pain

Infections: upper respiratory infection, fever, pharyngitis, bronchitis, oral herpes, herpes zoster, Malassezia, and Aspergillus

ROC curve analysis was performed for each of the three parameters for which correlation with gastrointestinal involvement was suggested. The analysis indicated the optimal cutoff for each parameter: 3.880 μg∙h/mL for AUC_0-0.5_, 24.360 μg/mL for Cmax, and 47.597 μg∙h/mL for AUC_0-12_ (Additional file 4A-C).

## Discussion

This study retrospectively investigated the electric medical records of 34 Japanese LN patients receiving MMF. MPA pharmacokinetic analysis revealed that renal function, serum albumin, concomitant PPI, and iron/magnesium oxide had significant effects (Fig. 2). As a PPK model, a two-compartment model with first-order elimination was developed in which the absorption process was best described by fixed 6 transit compartments (Fig. 3), and intra-individual variabilities were best described by a proportional residual error model. In the model, CLcr and serum albumin were significant covariates for CL (Table 2). For an LSS to be developed, single and double blood sampling strategies were examined by MAP-BE and MLR approaches. The results showed that the double sampling strategy of C_1_ and C_4_ by MLR best agreed with AUC_observed_ (*r*^*2*^ = 0.885). Of the single sampling strategies, the one at C_6_ by MAP-BE agreed best (*r*^*2*^ = 0.769) (Table 3).

MPA pharmacokinetics are reportedly affected by renal function. Reduced renal function can affect the MPA pharmacokinetics, leading to reduced elimination of and thereby higher exposure to MPA [4]. Because around 97% of MPA binds to albumin, hypoalbuminemia increases the free fraction of MPA, resulting in faster clearance of and thereby reduced exposure to MPA. In fact, low serum albumin concentrations have been reported to increase CL [4, 6]. In this study too, covariate analysis identified the effects of CLcr and serum albumin on CL. Although MPA pharmacokinetics could differ among populations [40], the estimated CL in our study was 13.15 L/h, comparable with those in the previous studies of Thai (14.5 L/h) [41] and Mexican (15.4 L/h) [10] patients with LN. Taken together, the MPA pharmacokinetics of the population in this study were similar in many ways to those in the previous studies: dose-normalized AUC_0-12_ increased significantly when renal function was damaged, but decreased significantly when serum albumin concentrations were low [40, 42]; and dose-normalized Cmax decreased significantly with concomitant PPI and iron/magnesium oxide (Fig. 2) [7, 43, 44]. These effects were taken into consideration in the first PPK model, in which creatinine clearance and serum albumin were covariates for MPA clearance and concomitant PPI and iron/magnesium oxide were covariates for V_1_ (Additional files 2, 5).

Absorption of MPA increases at low intragastric pH as the dissolubility of MMF increases [7, 45]. Although PPI was found to be a possible covariate of V_1_, histamine H2-receptor antagonist, which restrains gastric acid secretion, was not. A plausible speculation is that concurrent use of histamine H2-receptor antagonists does not affect pharmacokinetics overly much because these agents wear off as time passes, whereas PPIs impede irreversibly and provide lasting effects [46]. Besides PPI, iron/magnesium oxide decrease absorption of MPA as it forms a chelate with MMF [5, 47]. These facts may explain why concomitant PPI and iron/magnesium oxide were identified as possible covariates on V_1_.

PPI and iron/magnesium oxide, which are not always used for treating LN, were coadministered in 22 (64%) and 5 (14.7%) out of 34 patients, respectively, in this study. They were found to have significant effects in the pharmacokinetic analysis, and the PPK model including them, in addition to CLcr and serum albumin, as covariates significantly decreased OFV. Yet, suspecting overparameterization and considering evaluation adequacy and precision, we decided to exclude them from covariates for the final model. For confirmation of the decision, we examined LSSs from Bayesian estimation using the PPK model adding covariates of PPI and iron/magnesium oxide to the final model. As with the final model, best were the LSSs of C_6_ in single sampling and of C_6_ and C_8_ in double sampling (Additional file 6 and Table 3). Taking into consideration the same results in Bayesian estimation and the fact that they are not always necessary for treating LN, we found it appropriate the decision to exclude concomitant PPI and iron /magnesium oxide from covariates.

The PPK model with 6 transit compartments in this study offered a good description of the absorption process. The model did not, however, improve with the addition of EHC, which causes the second peak in MPA concentration. In a previous study, EHC was reported to account for as much as 40% of MPA AUC_0-12_ [4]. In this study, the second peak was seen in all but one patient, although peak values varied (Fig. 1A). Nevertheless, the EHC model did not significantly improve model fit. The failure of the EHC model may be because of the small number of patients, and also because of the lack of data on MPAG. MPA metabolizes into MPAG in the liver, and MPAG is thus involved in EHC [2]. In this study, however, MPAG concentrations were unknown and thus not taken into consideration. If the EHC model offered improved model fit, the prediction accuracy by MPA-BE would have been better.

As we mentioned earlier, AUC_0-12_ of MPA is reported to be well associated with clinical outcomes [19, 20] and is considered valuable for adjusting MMF dosage. As AUC_0-12_ requires frequent blood sampling, possible surrogate markers such as MMF dose and trough concentration have been examined [31]. In this study, MMF dose did not correlate with MPA AUC_0-12_ (*r*^2^ = 0.14). Whereas MPA concentration at C_0_ correlated with MPA AUC_0-12_ (*r*^2^ = 0.79) (Additional file 1), RMSPE was beyond the clinically acceptable range of 25% in evaluating the prediction accuracy of C_0_ single blood sampling strategy by MLR. These results suggest that either MMF dose or C_0_ MPA concentration may not work as surrogate markers for MPA AUC_0-12_. Further, no single sampling strategy by MLR showed RMSPE < 25%, suggesting that MPA concentration at any single time point is less likely usable as a surrogate marker. On the other hand, Bayesian estimation using the PPK model may allow a single blood sampling to predict AUC_0-12_ with sufficient accuracy. In this study, the best 2 strategies of C_6_ and C_8_ by MAP-BE were good enough for clinical use (Table 3).

When double blood sampling is available, the prediction accuracy of LSS naturally increases. The best 4 strategies by both MAP-BE and MLR, except for double sampling at C_1_ and C_3_, and double sampling at C_1_ and C_4_ by MAP-BE, were good enough for clinical use in this study. Although the best was the double sampling strategy of C_1_ and C_4_ by MLR, an LSS from MAP-BE may be preferable because of a few benefits. The MAP-BE approach might better estimate AUC_0-0.5_ and C_max_, which this study demonstrated were associated with gastrointestinal involvement (Table 4), especially when only one or two blood samples are available. An LSS by MAP-BE would thus have a better potential of facilitating dose adjustment in consideration of both effectiveness and tolerability. In addition, estimation by MAP-BE can be flexible in terms of blood sampling timing, making LSSs by MAP-BE more practical from the clinical perspective [40]. The accuracy of MAP-BE estimation would increase if the study size is larger and the PPK model improves.

A plausible application of this study to clinical practice would be to determine the initial dosing using the PPK model and then adjust the dosage based on the Bayesian estimation using the measured values so that AUC_0-12_ would be within the target range of 30–45 μg∙h/mL. AUC_0-0.5_ < 6.880 μg∙h/mL and Cmax < 24.360 μg/mL would be desirable for minimizing gastrointestinal symptoms.

This study showed some limitations. First, this study did not fully examine all factors that may affect MPA pharmacokinetics. Prednisolone was administered in all patients and NSAIDs in only three patients: for these medications, comparisons were therefore impossible. Data on diet, which can reportedly affect MPA pharmacokinetics [47], were unavailable, as were data on pharmacogenetics. Secondly, this study did not take free MPA concentration into consideration, because the data were not available. In that free drug is therapeutically active, it would be desirable to evaluate free concentration in MPA. Measuring free drug concentration, though it may be still difficult in clinical practice, should be done in a future study. As another limitation, external validation of the PPK model was not conducted due to the small number of patients. Validation from a larger study is needed in the future. Especially, further examination is warranted in a study with a larger number of patients receiving PPI and iron/magnesium oxide. Finally, the study did not investigate the effectiveness of MMF, which should also be a subject for future study.

## Conclusion

This study developed LSSs for Japanese LN patients receiving MMF so that the optimal MMF dosage could be individually determined based on estimated AUC_0-12_. When blood samples are taken twice, at 1 and 4 h after MMF administration, a strategy using the MLR approach would work best. When only a single blood sampling is allowed, however, a strategy using the MAP-BE approach with the PPK model developed in this study would be best with blood sampling at 6 h after administration. Although further studies are warranted, the LSS, whether single or double sampling, would facilitate safer, more effective, and individualized therapies for Japanese LN patients.

## Supplementary Information

**Additional file 1. **Correlations of MPA AUC_0-12_ with Dosage (A) and C0 (B).

**Additional file 2.  **Parameter estimates and bootstrap results of the PPK model that included PPI and iron/magnesium oxide in covariates.

**Additional file 3. **Scatter plots on logarithmic scale of observed versus predicted MPA concentrations.

**Additional file 4. **Receiver operating characteristic curve (ROC) analysis of associations between gastrointestinal involvement and pharmacokinetic parameters.

**Additional file 5. **Concentration versus time, goodness-of-fit plots of the PPK final model, and a visual predictive check for the final model.

**Additional file 6. **Best performing single and double sampling strategies for estimation of AUC_0-12_ of MPA by Bayesian estimation using the PPK model that included PPI and iron/magnesium oxide in covariates.

## Data Availability

Not applicable.

## Abbreviations

Alb: Serum albumin
ALT: Alanine aminotransferase
AST: Aspartate aminotransferase
AUC: Area under the concentration–time curve
AUC_0-12_: 12-Hour area under the concentration–time curve
CI: Confidence interval
CL: Central compartment clearance
CLcr: Creatinine clearance
CRP: C-reactive protein
eGFR: Estimated glomerular filtration rate
EHC: Enterohepatic recirculation
F: Bioavailability
FOCE ELS: First order conditional estimation-extended least squares
IIV: Interindividual variability
IMPDH: Inosine 5’-monophosphate dehydrogenase
IQR: Interquartile range
Ka: Absorption rate constant
Ktr: Rate constant of transit compartment
LLCI: Lower limit of the 95% confidence interval
LN: Lupus nephritis
LSS: Limited sampling strategies
MAP-BE: Maximum a posteriori Bayesian estimation
MLR: Multiple linear regression
MMF: Mycophenolate mofetil
MPA: Mycophenolic acid
MPAG: Mycophenolic acid glucuronide
MPE: Mean prediction error
MTT: Mean transit time
NSAID: Non-steroidal anti-inflammatory drug
OFV: Objective function value
pcVPC: Prediction-corrected visual predictive check
PPI: Proton pump inhibitor
PPK model: Population pharmacokinetic model
Q: Inter-compartmental clearance
RMSPE: Root mean squared percentage error
ROC: Receiver operating characteristic
RSE: Relative standard error
SLE: Systemic lupus erythematosus
TBIL: Total bilirubin
TDM: Therapeutic drug monitoring
ULCI: Upper limit of the 95% confidence interval
UPC: Urine protein/creatinine ratio
V1: Central volume of distribution
V2: Peripheral volume of distribution
