# Supplementary material for: Population pharmacokinetics and limited sampling strategy for therapeutic drug monitoring of mycophenolate mofetil in Japanese patients with lupus nephritis
Source: J Pharm Health Care Sci. 2023 Jan 9;9:1. doi: 10.1186/s40780-022-00271-w (PMC9830922; doi:10.1186/s40780-022-00271-w)
Supplement: Supplementary file 1 — Additional file 1. Correlations of MPA AUC0-12 with Dosage (A) and C0 (B). [file 40780_2022_271_MOESM1_ESM.docx]

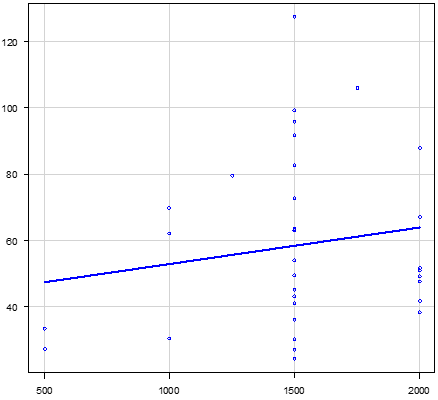

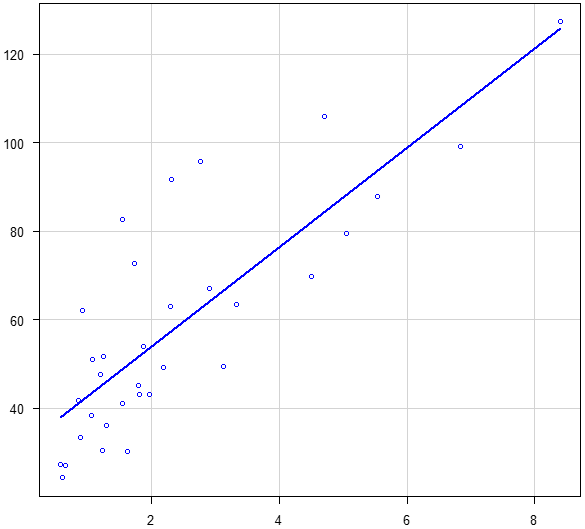


*r*^2^ = 0.14 (*p* = 0.43)

Dosage (mg)

C_0_ (㎍/mL)

AUC (μg∙h/mL)

**A**

**B**

AUC (μg∙h/mL)

*r*^2^ = 0.79 (*p* < 0.01)

**Additional file 1** Correlations of MPA AUC_0-12_ with Dosage (A) and C_0_ (B)
