# Supplementary material for: Population pharmacokinetics and limited sampling strategy for therapeutic drug monitoring of mycophenolate mofetil in Japanese patients with lupus nephritis
Source: J Pharm Health Care Sci. 2023 Jan 9;9:1. doi: 10.1186/s40780-022-00271-w (PMC9830922; doi:10.1186/s40780-022-00271-w)
Supplement: Supplementary file 2 — Additional file 2. Parameter estimates and bootstrap results of the PPK model that included PPI and iron/magnesium oxide in covariates. [file 40780_2022_271_MOESM2_ESM.docx]

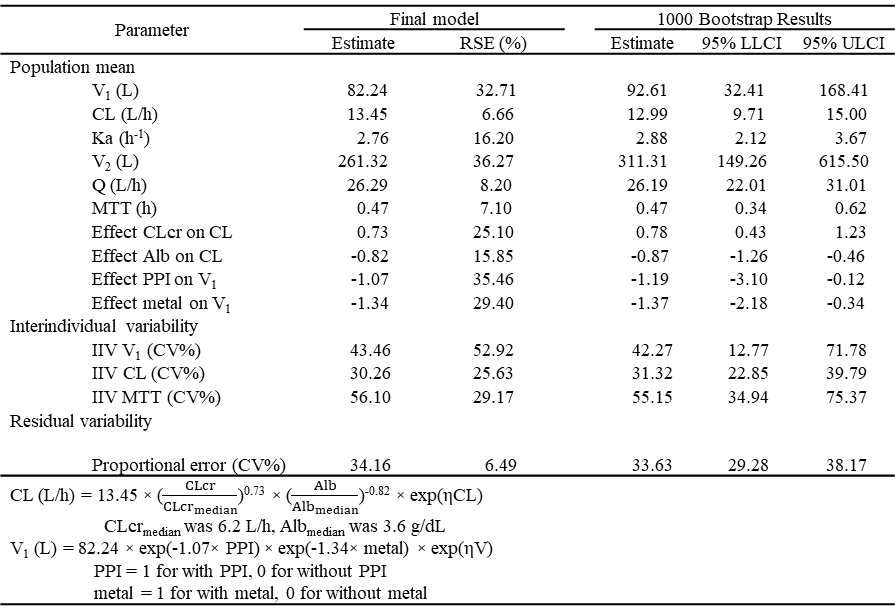


**Additional file 2** Parameter estimates and bootstrap results of the PPK model that included PPI and iron/magnesium oxide in covariates.

RSE, relative standard error; 95% LLCI, lower limit of the 95% confidence interval; 95% ULCI, upper limit of the 95% confidence interval; V_1_, central volume of distribution; CL, central compartment clearance; Ka, absorption rate constant; V_2_ peripheral volume of distribution; Q, inter-compartmental clearance: MTT, mean transit time; CLcr, creatinine clearance; Alb, serum albumin; IIV, interindividual variability; metal, iron/magnesium oxide
