# Supplementary material for: Population pharmacokinetics and limited sampling strategy for therapeutic drug monitoring of mycophenolate mofetil in Japanese patients with lupus nephritis
Source: J Pharm Health Care Sci. 2023 Jan 9;9:1. doi: 10.1186/s40780-022-00271-w (PMC9830922; doi:10.1186/s40780-022-00271-w)
Supplement: Supplementary file 3 — Additional file 3. Scatter plots on logarithmic scale of observed versus predicted MPA concentrations. [file 40780_2022_271_MOESM3_ESM.docx]

**
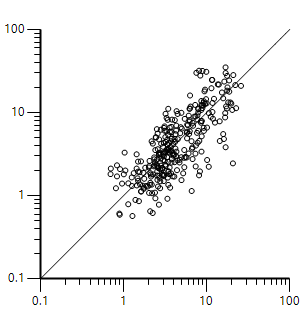
**

Observations (μg/mL)

Individual predictions (μg/mL)

**Additional file 3** Scatter plots on logarithmic scale of observed versus predicted MPA concentrations
