# Supplementary material for: Population pharmacokinetics and limited sampling strategy for therapeutic drug monitoring of mycophenolate mofetil in Japanese patients with lupus nephritis
Source: J Pharm Health Care Sci. 2023 Jan 9;9:1. doi: 10.1186/s40780-022-00271-w (PMC9830922; doi:10.1186/s40780-022-00271-w)
Supplement: Supplementary file 4 — Additional file 4. Receiver operating characteristic curve (ROC) analysis of associations between gastrointestinal involvement and pharmacokinetic parameters. [file 40780_2022_271_MOESM4_ESM.docx]

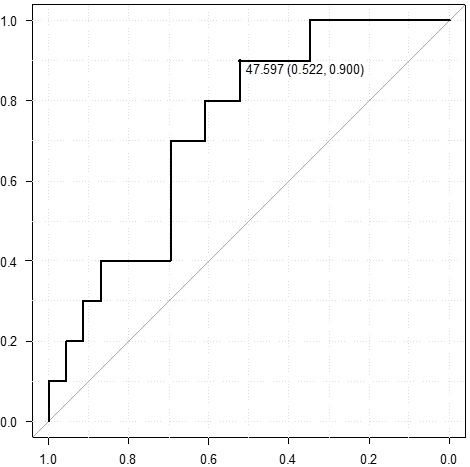

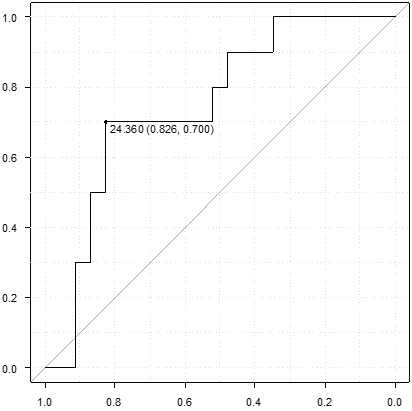

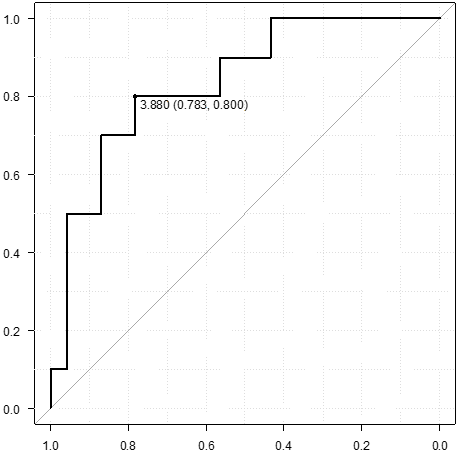


47.597

24.360

3.880

AUC_0-12_ (μg∙h/mL)

ROC-AUC = 0.730

Cutoff value 47.597

Cmax (μg/mL)

ROC-AUC = 0.748

Cutoff value 24.360

AUC_0-0.5_ (μg∙h/mL)

ROC-AUC = 0.835

Cutoff value 3.880

**C**

**B**

**A**

Sensitivity

Specificity

Specificity

Sensitivity

Specificity

Sensitivity

**Additional file 4** Receiver operating characteristic curve (ROC) analysis of associations between gastrointestinal involvement and pharmacokinetic parameters.

Significant classification models (p < 0.05) obtained by ROC analysis to discriminate between patients with and without gastrointestinal involvement by AUC_0-0.5_ (**A**), Cmax (**B**) and AUC_0-12_ (**C**). The reference line indicates no discrimination between groups. Proposed cutoff and ROC-AUC values are presented
