# Supplementary material for: Population pharmacokinetics and limited sampling strategy for therapeutic drug monitoring of mycophenolate mofetil in Japanese patients with lupus nephritis
Source: J Pharm Health Care Sci. 2023 Jan 9;9:1. doi: 10.1186/s40780-022-00271-w (PMC9830922; doi:10.1186/s40780-022-00271-w)
Supplement: Supplementary file 5 — Additional file 5. Concentration versus time, goodness-of-fit plots of the PPK final model, and a visual predictive check for the final model. [file 40780_2022_271_MOESM5_ESM.docx]

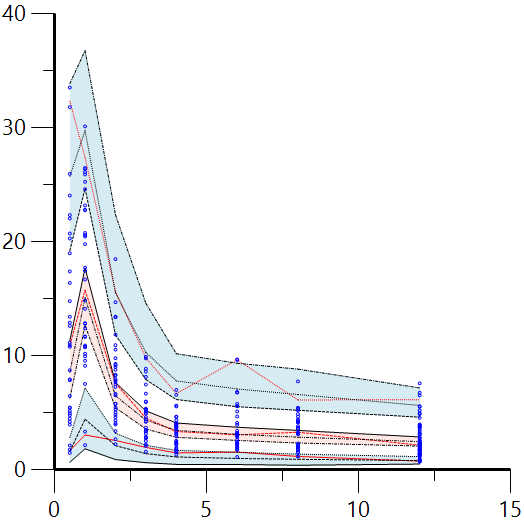

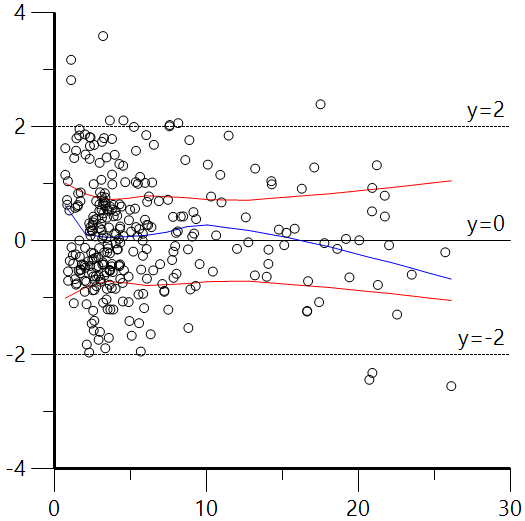

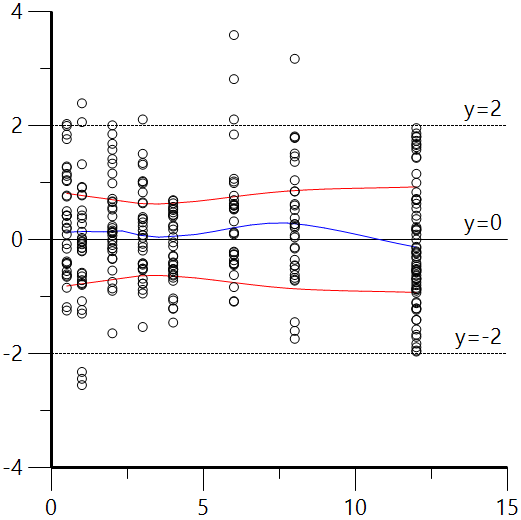

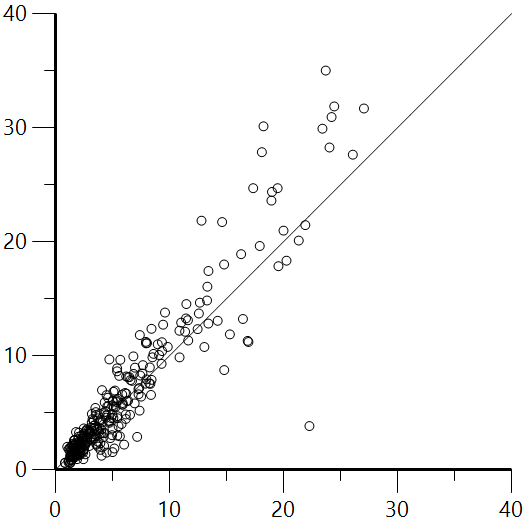

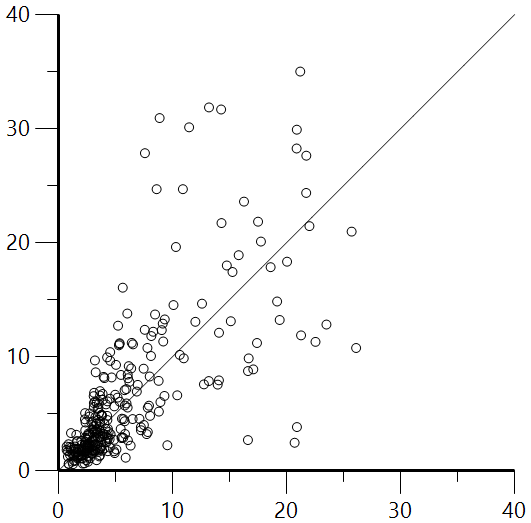

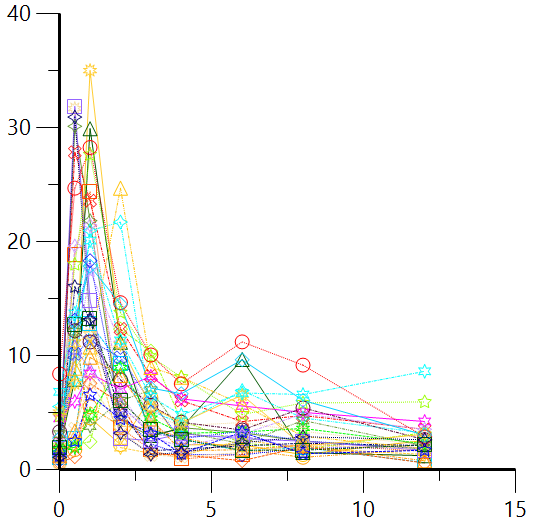


**Additional file 5** Concentration versus time, goodness-of-fit plots of the PPK final model, and a visual predictive check for the final model.

A) Concentration versus time profiles of MPA after administration. B–E) Goodness-of-fit plots of the final model. B) Scatter plots of observed concentrations versus individual predicted concentrations. C) Observed concentrations versus population-predicted concentrations. D) Conditional weighted residuals versus time since the dose. E) Conditional weighted residuals versus population-predicted concentrations. F) Prediction-corrected visual predictive check for the final model, where blue circles indicate observed concentrations, red lines represent the observed median and observed 5% and 95% quantiles, and shaded regions indicate 90%CIs for the simulations (orange regions indicate median, while blue regions indicate the 5% and 95% quantiles)

Population predictions (μg/mL)

Individual predictions (μg/mL)

Observations (μg/mL)

Time since the dose (h)

Conditional weighted residuals

Conditional weighted residuals

Prediction-corrected observations

**B**

**C**

**D**

**E**

**F**

Time since the dose (h)

Observations (μg/mL)

Population predictions (μg/mL)

Concentration (μg/mL)

Time since the dose (h)

**A**
