# Supplementary material for: Population pharmacokinetics and limited sampling strategy for therapeutic drug monitoring of mycophenolate mofetil in Japanese patients with lupus nephritis
Source: J Pharm Health Care Sci. 2023 Jan 9;9:1. doi: 10.1186/s40780-022-00271-w (PMC9830922; doi:10.1186/s40780-022-00271-w)
Supplement: Supplementary file 6 — Additional file 6. Best performing single and double sampling strategies for estimation of AUC0-12 of MPA by Bayesian estimation using the PPK model that included PPI and iron/magnesium oxide in covariates. [file 40780_2022_271_MOESM6_ESM.docx]

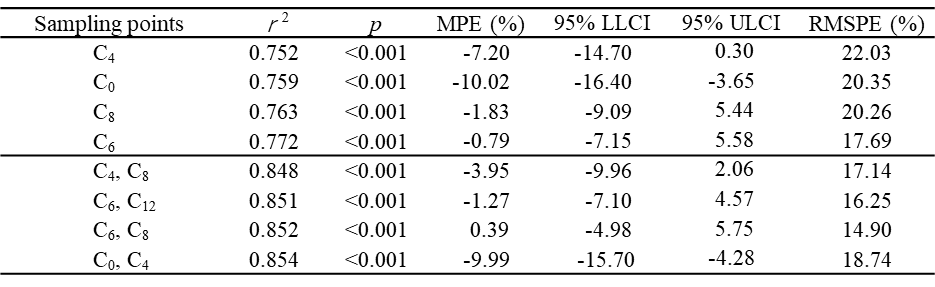


**Additional file 6** Best performing single and double sampling strategies for estimation of AUC_0-12_ of MPA by Bayesian estimation using the PPK model that included PPI and iron/magnesium oxide in covariates.

MPE, mean prediction error; 95% LLCI, lower limit of the 95% confidence interval of MPE; 95% ULCI, upper limit of the 95% confidence interval of MPE; RMSPE, root mean squared percentage error
